# Can statin preventative treatment inform geroscience‐guided therapeutics?

**DOI:** 10.1111/acel.13998

**Published:** 2023-10-13

**Authors:** Giovanni Guaraldi, Kristine M. Erlandson, Jovana Milic, Alan L. Landay, Monty A. Montano

**Affiliations:** ^1^ Modena HIV Metabolic Clinic University of Modena and Reggio Emilia Modena Italy; ^2^ Department of Surgical, Medical, Dental and Morphological Sciences University of Modena and Reggio Emilia Modena Italy; ^3^ Division of Infectious Diseases, Department of Medicine University of Colorado‐Anshutz Medical Campus Aurora Colorado USA; ^4^ Department of Internal Medicine Rush University Chicago Illinois USA; ^5^ Department of Medicine Harvard Medical School Boston Massachusetts USA

**Keywords:** geroscience, HIV, statin

## Abstract

Potential senotherapeutic effect of statins may lead to prevention and reduction of frailty.

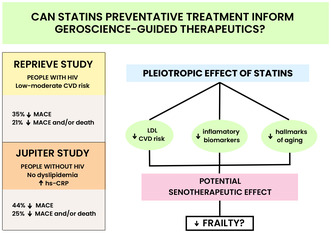

People with HIV (PWH) develop cardiovascular disease (CVD) at a significantly younger age than the general population, and in a context of multimorbid burden that is consistent with an accelerated aging phenotype (Grinspoon et al., 2023). The Randomized Trial to Prevent Vascular Events in HIV (REPRIEVE) study took center stage of the 2023 International AIDS Society (IAS) meeting in Brisbane, Australia, sharing findings that treatment with pitavastatin reduced the risk of major adverse cardiovascular events (MACE) compared with placebo in PWH without pre‐existing CVD (Grinspoon et al., 2023). Among 7769 persons with PWH (median age of 50 years) with low‐to‐moderate risk of atherosclerotic CVD, pitavastatin at a dose of 4 mg daily was associated with 35% lower risk of MACE and 21% lower risk of MACE and/or death over a median follow‐up of 5.1 years compared to placebo (Grinspoon et al., 2023). The effect of pitavastatin on first MACE was consistent among different subgroups, particularly in PWH with ART duration >10 years, nadir CD4 cell count <200 cell/μL, and current CD4 cell count >500 cell/μL (Grinspoon et al., 2023).

The REPRIEVE study builds on data from a previous notable primary‐prevention study involving statins, that is, rosuvastatin in the landmark JUPITER study (Ridker et al., 2008), which evaluated 17,802 healthy (median age of 66 years) adults without HIV infection or dyslipidemia, but with elevated high‐sensitivity C‐reactive protein (hs‐CRP), an inflammatory biomarker that predicts vascular events. Over a median follow‐up of 1.9 years, there was a significant reduction in cardiovascular events and reduction in hs‐CRP levels. Although hs‐CRP levels have not yet been reported from the REPRIEVE study, prior studies indicate that inflammatory biomarkers such as hs‐CRP, IL‐6 remain elevated in PWH despite effective ART but decline in response to statin therapy (Funderburg et al., 2014).

These trial results support a growing appreciation for the pleiotropic role of statins beyond reduction of plasma low‐density lipoprotein (LDL) levels. Statins also prevent plaque formation and prevent formation of cholesterol crystals that directly activate the NLRP3 inflammasome, a key mediator of inflammation (Koushki et al., 2021) and potentially also neurocognitive decline (Ising et al., 2019). Furthermore, given that lipophilic statins are widely distributed across tissues, whereas hydrophilic statins appear to be liver specific, carefully designed mechanistic studies that differentiate statin function are needed (Koushki et al., 2021).

These landmark prevention studies, considered together (pitavastatin in REPRIEVE and rosuvastatin in JUPITER) may inform geroprotective strategies—because CVD tends to be a comorbid condition (accelerated/accentuated by HIV infection) with advancing age that is potentially driven by a finite set of common aging‐related pathways, for example, hallmarks (Burch et al., 2014; López‐Otín et al., 2013; Toribio et al., 2017; Toribio et al., 2018). Whether the observed reduction in LDL within REPRIEVE and reduction in inflammatory biomarkers in JUPITER have common antecedent molecular drivers will be an exciting next chapter in these investigations.

One attractive common hallmark targeted by statins may be senescence. Indeed, we propose that statins may be considered geroprotective as a senotherapeutic agent, targeting senescence, either as senolytics (promote the clearance of senescent cells) or as senomorphics (block proliferation of a senescence‐associated secretory phenotype, SASP) (Coppé et al., 2008; Matsubayashi et al., 2023). Because cellular senescence underlies many age‐related conditions, senotherapy represents a promising approach for mitigating aging‐related conditions (e.g., CVD, metabolic disease, frailty, dementia). Statins may be geroprotective via a reduction of inflammation, secondary to lipid lowering, or possibly through a direct impact on key hallmarks of aging. Indeed, several studies in the general population suggest that statins influence hallmarks of aging. For example, statins appear to improve endothelial function through alteration of epigenetic pathways (Liuzzo & Pedicino, 2023) and telomere attrition (Nose et al., 2023). Interestingly, the American College of Cardiology has recently suggested to consider the impact of geroscience on therapeutic strategies for older adult with cardiovascular disease (Forman et al., 2023).

Notably, however, statins have also been linked to adverse effects, including an increased risk for metabolic dysfunction (Abbasi et al., 2021; Bai et al., 2023; Centers for Disease Control and Pre‐ vention, n.d.; Mansi et al., 2023; Sattar et al., 2010), albeit only minimally increasing glucose in the REPRIEVE study. The impact of statins on other pathways, may differ by population, dose and tissue effects. For example, statins may improve cardiovascular function following infarct through a direct impact on mitochondrial function (Bland et al., 2022). While myositis and myopathy are reported side effects of statins, these adverse outcomes tend to be rare. Nevertheless, their presence raises questions regarding the holistic health benefit‐to‐burden, and underscores the gap in our understanding of mechanism of action for statins. Notably, in the context of HIV, a recent clinical trial reported differences based on biological sex in statin‐mediated protection in loss of muscle mass, strength, and physical function (Cárdenas et al., 2023).

Notably in the REPRIEVE trial, myopathy related symptoms were marginally increased (2.3% vs. 1.4% in the placebo arm) while no significant differences in myopathy related symptoms were reported in the JUPITER trial (Ridker et al., 2008). Collectively, the benefits of reducing cardiometabolic, inflammatory biomarkers, and potentially SASP may outweigh the slight increased risk of myopathy‐related symptoms. This is especially significant given the linkage between inflammation and SASP with functional decline (Cesari et al., 2004) and frailty risk (Ferrucci & Fabbri, 2018).

Frailty, a condition characterized by critical exhaustion of functional reserve and resilience with aging may provide an opportunity to measure the impact of statins on the aging process (Afilalo et al., 2014; Forman et al., 2018). Frailty as a construct is a composite measure of vulnerability to stressors and the net result of competing forces, with some acting as gero‐protectors others as gero‐inducers. Ultimately, an imbalance of homeostatic reserves against stressors, expose an individual to a higher risk of negative outcomes, including falls, disability, comorbidities, cognitive impairment, and death (Rockwood & Mitnitski, 2007). Developing function‐promoting interventions that delay or prevent the onset of frailty may improve both healthspan and lifespan and is critically relevant in the context of the multimorbid burden observed in PWH (Montano et al., 2022).

People with HIV appear to experience earlier onset and greater prevalence of frailty, particularly among people with lower CD4 count and higher HIV‐1 RNA (Desquilbet et al., 2007). With the earlier initiation of more effective and less toxic antiretroviral therapy, we have demonstrated a decrease in the prevalence of frailty among PWH aged 50 years, but a 3‐fold increase among those 75 years of age with the Modena HIV Metabolic clinic, suggesting a potential that in this specific population, contemporary ART may be geroprotective (Guaraldi et al., 2019). Importantly, CVD is consistently an important risk factor for frailty across multiple cohorts (Kelly et al., 2019; Kuniholm et al., 2022).

The findings from REPRIEVE suggest that decreasing CVD risk with statins may have added benefits through alteration of numerous aging “hallmarks”, ultimately slowing the progression of geriatric syndromes such as frailty. A substudy of REPRIEVE (PREPARE NCT04221295) collected objective measures of physical function, muscle area, muscle density, and the frailty phenotype among a subset of REPRIEVE participants (Umbleja et al., 2020). The relatively young age of the cohort and low prevalence of baseline frailty may; however, limit the ability to detect changes in some of these outcomes typically ascertained in cohorts of individuals 65 years or older (Umbleja et al., 2020).

Finally, in the light of the REPRIEVE findings, we anticipate that guidelines will soon reflect earlier initiation of statin therapy in PWH, a dramatic change from the relatively low current prescription rates for statins (Jaschinski et al., n.d.). This is a critical area of clinical roll‐out, given that by the end of 2030 about 70% of PWH will be more than 50 years old and 78% will have CVD (Smit et al., 2015). However, these estimations might not be generalizable to all geographical areas. Indeed, in REPRIEVE, the authors noted that other CV risk factors had higher prevalence in high income countries (e.g., greater BMI, smoking, family history of CVD) (Grinspoon et al., 2023). As these risks are expected to increase with the changing epidemic of HIV, the growing obesity epidemic among people with HIV in low and middle‐income countries, and the weight changes observed with modern ART in comparison to older regimens, the cardioprotective effects of statin therapy could be even greater in the coming years. Because CVD is often comorbid with other aging‐related conditions such as frailty, the early use of statins may influence outcomes for other age‐related comorbid conditions. Indeed, the REPRIEVE study may represent a breakthrough both in HIV medicine, as well as in the broader field of geroscience‐guided therapeutics (Forman et al., 2023).

## AUTHOR CONTRIBUTIONS

Giovanni Guaraldi and Monty A. Montano conceptualized and designed the manuscript. Giovanni Guaraldi, Kristine M. Erlandson, Jovana Milic, Alan L. Landay, and Monty A. Montano wrote and revised the manuscript. Giovanni Guaraldi, Kristine M. Erlandson, Jovana Milic, Alan L. Landay, and Monty A. Montano did the supervision of the final version of the manuscript. All the authors contributed to discussion and revised the manuscript.

## FUNDING INFORMATION

The authors received no funding to produce this manuscript.

## CONFLICT OF INTEREST STATEMENT

GG received research grants and speaker honoraria from Gilead, ViiV, MERCK, and Jansen. GG attended advisory boards of Gilead, ViiV and MERCK. KME has received grant funding from Gilead and had participated in advisory boards for Gilead, ViiV, and Merck. All payments have been to the University of Colorado. JM received speaker honoraria from Gilead and ViiV. AL receives research funding from Abbott and Gilead. MM is Editor in Chief of Aging Cell (John Wiley and Sons).

## Data Availability

N/A
